# Overview of Computational Toxicology Methods Applied in Drug and Green Chemical Discovery

**DOI:** 10.3390/jox14040101

**Published:** 2024-12-04

**Authors:** Jose I. Bueso-Bordils, Gerardo M. Antón-Fos, Rafael Martín-Algarra, Pedro A. Alemán-López

**Affiliations:** Pharmacy Department, CEU Cardenal Herrera University, CEU Universities C/Ramón y Cajal s/n, Alfara del Patriarca, 46115 Valencia, Spain; ganton@uchceu.es (G.M.A.-F.); rmartin@uchceu.es (R.M.-A.); paleman@uchceu.es (P.A.A.-L.)

**Keywords:** computational toxicology, machine learning, deep learning, quantitative structure–activity relationship (QSAR), environmental toxicology

## Abstract

In the field of computational chemistry, computer models are quickly and cheaply constructed to predict toxicology hazards and results, with no need for test material or animals as these computational predictions are often based on physicochemical properties of chemical structures. Multiple methodologies are employed to support in silico assessments based on machine learning (ML) and deep learning (DL). This review introduces the development of computational toxicology, focusing on ML and DL and emphasizing their importance in the field of toxicology. A fine balance between target potency, selectivity, absorption, distribution, metabolism, excretion, toxicity (ADMET) and clinical safety properties should be achieved to discover a potential new drug. It is advantageous to perform virtual predictions as early as possible in drug development processes, even before a molecule is synthesized. Currently, there are numerous commercially available and free web-based programs for toxicity prediction, which can be used to construct various predictive models. The key features of the QSAR method are also outlined, and the selection of appropriate physicochemical descriptors is a prerequisite for robust predictions. In addition, examples of open-source tools applied to toxicity prediction are included, as well as examples of the application of different computational methods for the prediction of toxicity in drug design and environmental toxicology.

## 1. Introduction

With the rapid growth of computer technology and the increasing availability of biochemical activity data, computational tools have come a long way in chemistry and life sciences. With the help of computer technology, scientists can predict the results of chemical experiments by creating several realistic models, producing a new discipline of computational toxicology [1].

Computational toxicology is a broad term, which covers computer-aided toxicology, computational modeling technology, contact modeling, physiological-based kinetic modeling and dose–response modeling. The goal of all these disciplines is to combine information and data to investigate adverse health effects of chemicals or drugs by constructing mathematical models. The early animal toxicity assays, which used lots of material and financial resources, were time-consuming and labor-intensive and were even met with strong opposition from the animal rights association and public pressure [2]. Currently, there are more than 85,000 chemicals registered with the Environmental Protection Agency (EPA) under the Toxic Substances Control Act, but few of them have been evaluated for potential toxicity [3].

Hopefully, toxicity tests will be separated from animal models and rely on in vitro methods to study or describe toxicity mechanisms. To some extent, computational toxicology has become a complementary method to traditional toxicity experimental assays. The toxicity prediction model of compounds established by computer technology, based on the toxicological properties and toxicity mechanism of compounds, can be predicted without experiment [4]. The need to use data from multiple methods to carry out a safety assessment has led to the concept of Integrated Approaches to Testing and Assessment (IATAs). Within an IATA, data from various information sources are evaluated and integrated to draw conclusions on the hazard and/or risk of chemicals [5]. In fact, a complete framework called the “adverse outcome pathway” (AOP) has been proposed in the field of toxicology to summarize the hypothetical key biological events that lead to adverse effects. This has been a major milestone in toxicology, and numerous lines of research on the use of computational methods to predict toxicity are growing fast [6].

Clinical efficacy and safety are two of the most common reasons for drug failure. Computational tools for predicting drug toxicity began in the early 2000s and can now be used in every phase of drug design. In the field of drug discovery, the best strategies are to strike a reasonable balance between in vivo, in vitro, and computational pharmacology and toxicology predictions are employed as early as possible to evaluate the activity and safety of lead compounds [7]. Mathematical principles and advanced computer models are used to assess the harm of drugs to human beings and the environment. The advanced information science, high-throughput screening (HTS) and other technical principles can be used to establish a stable computing tool to screen the potential toxicity of large-scale libraries of compounds to assess the harm of drugs and chemicals to humans and the environment [8].

In the current review, we aim to summarize the research methods and the application of machine learning and deep learning in environmental research, as well as the research background and significance of computational toxicology.

## 2. Machine Learning and Deep Learning in Computational Toxicology

Artificial intelligence (AI) is the ability of computer systems to perform tasks that typically require human intelligence. This includes visual perception, speech recognition, learning from experiences, language translation, recognizing patterns, and making decisions [9].

Machine learning (ML) is a subset of AI that uses statistical methods to enable machines to improve with experience. This is particularly useful in life sciences, where vast amounts of data are generated [10]. Deep learning (DL) is, in turn, a subset of ML, which is continuously and rapidly expanding to constitute its own field of AI. DL models are composed of multiple processing layers for learning representations of data with multiple levels of abstraction [11].

AI can significantly speed up data processing and analysis in bioinformatics, providing insights into disease mechanisms, drug targets, toxicological effects, etc. AI and toxicology have evolved as distinct scientific disciplines over the past several decades. While toxicology has its roots in traditional observational studies, AI is a rapidly developing field with tremendous impact on various domains of the life sciences, which has created new opportunities for applying AI techniques. The synergistic integration of these two disciplines can pave the way for the next generation of predictive, mechanistic, and data-driven safety science [12].

Currently, the role of ML methods in computational toxicology has become very important with the increasing availability of diverse toxicology data streams, which has led to the demand for complex data analysis algorithms. ML is a discipline which uses calculation methods and empirical methods to improve the performance of the target itself. Most ML methods are supervised methods that analyze the descriptors of chemical compounds in a training set to construct a model. In addition, AI is revolutionizing the drug discovery process by predicting how different drugs will interact with targets in the body. ML algorithms can process massive amounts of data on molecular structures and biological processes to help predict drug outcomes and side effects [13].

Quantitative structure–activity relationships (QSARs) can be generated using different programming tools and modeling software, which enable the application of ML methods to computational toxicology. Table 1 summarizes different software and web services that can be used to build QSAR models.

The creation of virtual combinatorial libraries (VCLs) is a crucial part in the early phases of the drug discovery process since these libraries are used to explore QSAR models to identify lead compound derivatives [23]. Useful tools that can be used to build VCLs are KNIME, RDKit, DataWarrior, and Reactor [24,25,26,27], which allow for the design of small chemically feasible molecules based on a list of generic reactions considering reaction rules.

Since biological, medical and chemical data have the characteristics of a high dimension, strong heterogeneity and complex redundant information, it is particularly prominent in the field of computational toxicology to use ML to mine necessary information and rules [28]. For example, many methods of computational chemistry and computational biology are involved in computer-aided drug design (CADD). The molecular structure of drugs is one of the influencing factors of drug activity, so it is necessary to use multiple regression analysis to find out the most important structural factors that may affect drug efficacy [29].

In the process of receptor-based drug molecular design, when the molecular docking score is used to determine whether a small molecule is an inhibitor or not, the receiver operating characteristic (ROC) curve can be used to find the best classification model considering the statistical significance [30,31]. There are simple examples of ML methods in drug design, and many methods have been successfully applied to computational toxicity prediction, such as an artificial neural network (ANN), hidden markov chain (HMC), support vector machine (SVM), decision tree (DT) and random forest (RF) [32].

DL is a subfield within ML that structures algorithms into layers to create an artificial neural network capable of learning and making intelligent decisions on its own. Due to its high popularity in recent years, neural networks with multiple hidden layers have developed into different, multi-level and abstract neural networks [33,34]. The active areas of DL include image processing, computer vision, speech recognition, natural language processing, and so on, which are innovative to some extent. It can be said that the innovation of the ANN structure, the rise of algorithms, the development of computer technology and the acquisition of large data sets have greatly promoted DL to become a powerful tool for structure–property–activity research and drug discovery [35,36,37]. DL can use an ANN with multiple layers of nonlinear processing units to model high-level abstractions contained in the data. The combination of simple functional groups into complex functional groups makes DL very suitable for computational toxicology.

Jeong and Choi analyzed the databases, molecular descriptors, fingerprints and algorithms considered in various studies. They found that the molecular access system and extended connectivity fingerprints are the most commonly used molecular descriptors for model development. RF is the most used algorithm among ML techniques, while the most used algorithm among DL techniques is a DNN [38].

For example, Zheng et al. screened 331 kinds of hemolytic saponins and 121 kinds of non-hemolytic saponins in their study to explore the prediction of saponins’ hemolytic toxicity by ML methods, including K-nearest neighbors (KNN), SVM, RF and gradient boosting machine (GBM), and all four models showed good performance [39]. Hemmerich et al. used ML methods to study the prediction of mitochondrial toxicity. The authors collected a data set of 5761 mitochondrial compounds and used RF, GBM and DL methods to construct their model. Since the data set was imbalanced, sensitivity, specificity, their balanced accuracy (BA) and positive predictive values were chosen to be calculated. BA can predict the average performance of the model. Finally, the BAs of RF, GBM and DL were 0.866, 0.894 and 0.895, respectively [40].

In this line of work, Mayr et al. established the DeepTox pipeline to apply DL to predict toxicity more properly and concluded that DL significantly outperforms other ML methods such as SVM, NB and HMC in a Tox21 data challenge [41]. The mechanism is explained by the activation of hidden neurons at different levels. In the process of developing neurons from a low level to a high level, the substructure of the characteristic-coding toxicological carrier in which neurons are located gradually becomes larger until it occupies the whole toxicological carrier. Therefore, deep neural networks (DNNs) can learn from complex toxicological characteristic data, which leads to a high prediction ability of toxicity [42].

More recently, Yang et al. designed perfluoroalkyl and polyfluoroalkyl substances (PFASs) by incorporating DL and molecular generative models. They performed virtual screening (VS) using MolHGT+ and found that the presence of the siloxane group and betaine fragment decreased both the bioaccumulation and hepatotoxicity of PFAS while maintaining a low surface tension. In addition, they generated a new group of PFASs using generative molecular models [43].

## 3. Computational Toxicity In Silico Methods

The in silico prediction of toxicological outcomes has become increasingly popular due to its expeditious and low-cost return of results. Currently, there are many commercially available and free web-based programs for toxicity prediction and a variety in silico methods developed to calculate the toxicity of chemicals [44,45]. A brief description of the main computational predictive methodologies is shown in Table 2.

The QSAR is a method to predict chemicals’ activity based on molecular descriptors. Pharmacokinetic (PK) models can calculate the amount of chemicals in different parts of the body by relating chemical concentration in tissues. Pharmacodynamic (PD) models correlate a biological response with the concentration of a chemical. Structural alerts (SAs) are chemical structures associated with toxicity. Read-across (RA) is a method to predict the unknown toxicity of a chemical based on similar chemicals with known toxicity [46].

Animal testing for the evaluation of toxicological risks of substances is increasingly questioned on ethical grounds. For this reason, the so-called new approach methodologies (NAMs) have been developed, covering a wide range of methods, in silico, in vitro and in chemico [47,48]. The present study tries to summarize the NAMs based on computational modeling and RA methods.

Computational models are generated quickly to predict toxicological hazards and results, with no need for test material or animals. Considering the wide variety of toxicity, endpoints and mechanism of action, computational toxicology comprises rule-based systems, molecular docking, pharmacophore modeling, quantum chemistry studies and structure–activity–properties models. There are two methods of computational toxicology: one is to calculate the chemical structure of the compounds themselves, and the other is to calculate the chemical structure based on the toxic targets. The former does not need to clarify the mechanism of toxicity but only needs to understand the structure of its compounds. This method can be divided into two branches: the statistical numerical method and the rule reasoning method. However, for the latter, it is necessary to understand the toxicological mechanism of compounds, as well as the toxic effects of toxic compounds and biological macromolecules, also known as the molecular mechanism method [49].

The main idea of computational toxicology is based on QSAR or quantitative structure–property relationships (QSPRs). The objective of the QSAR/QSPR methods is to link available data on various physicochemical properties and biological activities with the chemical structures of the compounds exhibiting such properties and to build mathematical models by means of molecular descriptors. The key features of QSAR/QSPR modeling are as follows [50]: (1) the prediction of a given response (activity, property or, as detailed later on, toxicity); (2) the replacement of or reduction in animal experimentation; (3) the virtual screening of data sets; (4) the establishment of a probable mechanistic interpretation; (5) the categorization of data (this objective is primarily useful in the assessment of chemical toxicity where data can be classified into different levels of hazard); (6) the optimization of lead molecules; and (7) the structural refinement of synthetic target molecules. Many research groups [51,52,53,54,55,56] are adopting this strategy, for instance, to repurpose drugs for which safety and toxicity data have been already collected from clinical assays, to use commercial compounds with new pharmacological activities.

The relationships between a physical property and a pharmacological activity (quantitative property–activity relationships, QPARs) have also been studied, such as cytotoxicity or the potency of local anesthetics and the octanol/water partition coefficient, as well as between the chain length or surface tension and narcosis. In these cases, the calculation of molecular descriptors is not necessary [57].

Several statistical methods are chosen to construct predictive models, such as regression analysis (RA), linear discriminant analysis (LDA), principal component analysis (PCA), cluster analysis (CA), a genetic algorithm (GA), an ANN, and so on.

## 4. Application of QSAR in Toxicity Prediction During Drug Design

QSAR modeling is a technique that allows the interdisciplinary exploration of knowledge on compounds, covering chemical, physical, biological and toxicological aspects. Likewise, it provides formalisms for the mathematical development of models based on chemical characteristics and the activity of structurally similar compounds. This context is defined by mathematical algorithms and provides a reasonable foundation for the creation of a prediction model. Computational toxicology was initially used in drug discovery and later found to play an important role in environmental science. The relationship between the molecular structure and toxicity of pharmaceutical drugs established by quantitative structure–toxicity relationship (QSTR) models is based on computational toxicology principles, which are finally applied and validated by unknown new compounds [58].

In this line, Galvez et al. studied the activity of general insecticides and insect growth regulators (IGRs) against *Anopheles* and *Culex*, both *Plasmodium falciparum* vectors. Insecticidal activity was expressed in different ways depending on the group: for general insecticides, activity was expressed as the “dose of active ingredient” (g/m^2^). For hormone analogs, toxicity was expressed as LC50, which is defined as the lethal concentration that causes a 50% inhibition of the development of larvae to adult mosquitoes, expressed in ppm. A discriminant model of antimalarial activity was obtained, capable of correctly classifying 96% of the studied compounds, demonstrating that the use of topological indices yields good results in predicting insecticide activity against malaria vectors [59].

It might be interesting to point out that in large libraries, a pre-screening process is usually carried out prior to the VS process, non-specific to the biological target, to eliminate structures that have non-drug properties. That is, they consider whether the molecule is biologically relevant in terms of the functional groups it has and its physical properties (drug-likeness). The first to observe this was Lipinski, and he described the so-called “rule of five”, which establishes that a molecule will have adequate oral absorption if it meets three of the four rules [60]. Other descriptors also used include the number of atoms, rotatable bonds and the electronic charge of the molecule [61].

Other authors have included other similar requirements such as the polar surface area (PSA), which has been used in medicinal chemistry for the optimization of a drug’s capability to permeate cells. For instance, Pajouhesh and Lenz correlated molecules with a PSA > 140 Å^2^ to a low ability at permeating cell membranes, whereas drugs with a PSA < 60 Å^2^ were completely absorbed [62]. Hitchcock and Pennington suggested a threshold of 90 Å^2^ for increasing the potential for blood–brain barrier penetration [63]. However, Hughes et al. established the “rule of 3/75”, which states that a compound with low ClogP/high topological PSA (TPSA) will be 2.5 times safer in in vivo assays, that is, when its ClogP < 3 and its TPSA > 75 [64].

The presence of certain functional groups that are not desirable, such as reactive moieties and known toxicophores (SAs), may lead to false positives due to reactivity or assay interference, which have long been noted as a problem in high-throughput screening (HTS) [63]. These SAs are applied during the pre-screening process, whose goal is to detect toxic or too unstable functional groups, which are usually removed from virtual libraries due to their reactivity or interferences produced in assays [65]. A representative list of such undesirable groups along with their screening liabilities [61,66,67,68,69] is shown in Table 3.

However, it is important to note that many known drug molecules contain SAs; there is also evidence indicating the formation of active metabolites as a causal factor for the toxicity of 62–69% of these molecules [70]. These data highlight that pre-filtering is used to reduce risk, but will also eliminate useful molecules from further consideration. As increasing amounts of assay data from different HTS are becoming publicly available, a clearer pattern of compounds and functional groups that tend to yield false positives is developing. This is becoming vital as non-specific potentially active molecules are likely to be over-represented from chemical vendors due to an increased likelihood that they will be ordered as derivatives of potential hits. While this may be acceptable in a screening hit, it would almost certainly have to be removed in the lead optimization process [61].

For instance, Duart et al. jointly used RA and LDA to produce an antihistamine activity prediction model [71]. Subsequently, an equation capable of predicting sedation was included, selecting compounds with theoretical antihistamine activity and no sedative effects [72]. These topological models were used to find new antihistamines through the creation of a virtual combinatorial library. Seven compounds were synthesized, six of which were active in vivo, and two of them exhibited greater activity than the reference compound, terfenadine [71]. Additionally, this same model was applied to databases of thousands of compounds consisting of pharmaceutical drugs and chemical reagents. Of these, eight compounds were tested in vivo, all of them were active, and three of them were also more active than terfenadine [72]. The fact that the model selected drugs with other therapeutic applications as theoretical actives is interesting since the search for other applications in known drugs is a common practice in the development of new drugs, saving time and resources in clinical and preclinical phases since their toxicity and pharmacokinetics have already been described [73].

M-Y. Wang et al. developed a combined in silico method to predict potential protein targets involved in cardiotoxicity induced by aconitine alkaloids. A protein–protein interaction (PPI) network was built using the STRING database to extract relevant protein interaction information related to aconitine cardiotoxicity. Calcium–calmodulin-dependent protein kinase II alpha (CAMK2A) and gamma (CAMK2G) were identified as potential targets. The study employed QSAR models, demonstrating internal robustness and high external predictive ability. Molecular dynamics simulations indicated that aconitine alkaloids exhibited binding stability with the CAMK2G receptor. This study helped guiding structural modifications of aconitine alkaloids and allowed a better understanding of the cardiotoxicity associated with structurally similar compounds [74].

ADMET (absorption, distribution, metabolism, excretion and toxicity) properties, which are predicted, are factors that are increasingly being included in the previous stages of VS to simultaneously optimize potency and pharmacokinetics. These models are particularly useful since knowing the ADMET profile of a molecule with theoretical activity allows a better selection of drug candidates since those with undesirable pharmacokinetic characteristics can be directly discarded.

In this line of work, Speck-Planche et al. developed two multifunction models to establish relationships between the chemical structure of compounds and their microbiological effect on *Escherichia coli* and *Pseudomonas* spp. by using structurally heterogeneous databases with over 20,000 molecules. In both cases, the models simultaneously predicted the antibacterial activity and the ADMET profile of the molecules. These models were validated by virtually predicting the properties of compounds whose activity against *E. coli* and *Pseudomonas* spp. (avarofloxacin and delafloxacin, respectively) were known [75,76].

## 5. QSAR Application to Environmental Toxicology

Nowadays, the emphasis of computational toxicology is placed in the field of environmental toxicology. Thus, the next step is to predict and evaluate the toxicity of potential environmental pollutants. QSAR-based computational toxicology is a new method in environmental toxicology [77]. Model performance can only be compared between studies when using the same data set. Table 4 lists four classic applications of computational toxicology in the environment.

The U.S. EPA launched the “Tox Cast” project in 2008 to identify suspicious environmental pollutants by using in vitro HTS approaches to seek a structure–effect relationship covering carcinogenicity development, reproductive toxicity, neurotoxicity and so on. Computational toxicology analyses of pollutants have been reported by using quantum chemistry, the molecular connectivity index method and other methods [82]. More recently, with the goal of developing higher-throughput in vitro systems and computational models to predict the response in humans and the environment, the “Tox21” project was launched as a partnership between the EPA, the National Toxicology Program (NTP) and the NIH’s Chemical Genomics Center (NCGC), and the US Food and Drug Administration (FDA). Since then, the project has analyzed nearly 10,000 chemicals and generated more than 100 million data points, all of which are publicly available [83].

Moreover, Schür et al. have recently developed ADORE, a comprehensive data set on acute aquatic toxicity in three relevant taxonomic groups (fish, crustaceans and algae). The data set includes ecotoxicological experiments and phylogenetic and species-specific data, as well as chemical properties and molecular representations [84].

In this line of work, Manzetti used RA and a neural network to establish a QSAR model to study the toxicity of halogenated polycyclic aromatic hydrocarbons, amines and nitrobenzene compounds to aquatic organisms [85].

A. Kumar et al. developed regression-based QSTR models to assess the toxicity of organic chemicals on three protozoan species (*Entosiphon sulcantum*, *Uronema parduczi*, and *Chilomonas paramecium*). Using three sets of chemical descriptors (ETA indices, non-ETA descriptors, and a combination of both), the models identified key structural features, such as non-polar characteristics, electronegativity, hydrogen bonding, π–π, and hydrophobic interactions, that influence toxicity. The validated models were applied to screen the DrugBank database for ecotoxicological properties and can be used for designing eco-friendly drugs, filling toxicity data gaps, and reducing hazardous chemicals in the environment [86].

Gita et al. used the green algae *Chlorella vulgaris* to study the toxicity of three textile dyes, including optilan yellow, drimarene blue and lanasyn brown. With an increase in dye concentration (0–50 mg/L), the inhibition rate increased from 50% to 80%. The results showed that the growth and biological productivity of microalgae decreased with the increase in dye concentration, among which drimarene blue had the greatest toxicity to microalgae, and other dyes had moderate toxicity [87].

Similarly, Grote et al. used toxicology-related algorithms to verify the feasibility of a method for qualitatively and quantitatively predicting the phototoxicity of *Scenedesmus vacuolatus* in green algae. The gap between the highest occupied molecular orbital and the lowest unoccupied molecular orbital of *Daphnia magna* (*D. magna*) was used as a qualitative indicator of the potential phototoxicity of the green algae *Scenedesmus vacuolatus* [88].

Duan et al. developed a full strategy based on a high-throughput experiment for mixture toxicity analysis, in which an RF algorithm was used to automatically screen fit parameters, with high learning efficiency, high precision and a strong generalization ability, to predict the acute toxic effects of compounds [89].

Chen et al. chose only six molecular descriptors to establish a QSAR model for 96 h PLC50 by an SVM and genetic algorithm based on 963 organic compounds with acute toxicity to fathead minnows. The best SVM model (r^2^ = 0.756) was satisfactory for the prediction of acute toxicity and verified by using both internal and external validations [90].

Based on the mutagenicity against *Salmonella typhimurium* strain TA100, Hao et al. used QSAR and classification models to predict the potential toxicity of nitroaromatic compounds, a class of important environmental organic pollutants. The best QSAR model exhibited reliable results based on E-DRAGON [91] and quantum chemistry descriptors, and the obtained statistical parameters were as follows: q^2^_loo_ = 0.950, r^2^ = 0.967, q^2^_test_ = 0.836, and r^2^_test_ = 0.843 [92]. The main issue concerning this study is that, although relatively recent, currently, E-Dragon seems to be an abandoned and no-operational platform, which certainly hinders the reproducibility of their findings.

Moreover, ionic liquids or ILs (salts with melting points below 100 °C that contain differentiated anions and cations) have also gained popularity. Considered “green solvents” due to their specificity and minimal release into the environment, the assessment of their toxicity to ecosystems has received considerable attention in recent years. The development of QSAR models for ILs can help in the search and design of suitable chemicals with a reduced toxicity profile [93,94]. There are studies that show the negative effect that ILs can have on the environment, as well as their toxicological effects on various microorganisms, with the aquatic environment the first to be addressed.

Namely, Das et al. applied connectivity descriptors combined with LDA and RA to capture the specific structural information of ILs responsible for their toxic manifestation to *V. fischeri*. The discriminant model was characterized by acceptable Wilk’s λ statistics, a pharmacological distribution diagram (PDD) assessment and ROC analysis parameters. The regression models were assessed according to the OECD guidelines, and the best model showed satisfactory external predictivity (r^2^_pred_ = 0.739). The toxicity of ILs to *V. fischeri* was found to be inversely related to molecular branching and size in both models [95].

Wang et al. measured the toxicity of 24 bromide-based ionic liquids (Br-ILs) towards *V. fischeri* and *D. magna* and established a good QSAR model with correlation coefficients (r^2^) of 0.954 and 0.895 for *V. fischeri* and *D. magna*, respectively. The model of *V. fischeri* showed that Br-IL toxicity was inversely related to the energy of the lowest unoccupied molecular orbitals (ELUMOs). The model of *D. magna* suggested that Br-IL toxicity showed a positive correlation with the dipole moment (μ) [96].

K. Roy and R.N. Das developed predictive models of the toxicity of ILs towards *D. magna* from the chemical structure of 62 different ionic liquids using extended topochemical atom (ETA) indices as descriptors along with other topological and thermodynamic parameters that revealed how lipophilicity, branching and chain length influence toxicity [97].

By using these QSAR-based models, any untested new IL analogs falling within the defined applicability domain can be successfully predicted for their possible toxic effects. ILs may be ranked in terms of their toxicity and the amount of animal experimentation may also be reduced. All this physicochemical and structural information can be highly useful for designing new suitable ILs.

## 6. New Insights and Challenges for Computational Toxicity Prediction

There are still some issues to be overcome with traditional in vivo and in vitro toxicity tests, including both the fact that they can be laborious, time-consuming and highly expensive and the application of ethics to animal welfare. Computational toxicity prediction can compensate for the shortcomings of traditional approaches and can be successfully applied in the early stages of drug development. With the advancement of computational theory and molecular representation, numerous toxicity prediction models are developed and used either in a predictive sense or in a problem-solving setting. Although the theoretical prediction methods of toxicity have made remarkable progress, the challenges and difficulties are still quite great. The key problems lie in the lack of complete and effective toxicity data, low prediction accuracy of some models, and narrow application range of the models [98].

In addition, due to the lack of theoretical prediction methods of toxicity, there are not enough predictable material objects. However, due to the strong support of the scientific community and government departments, with the in-depth development of the 3R principle (replacement, reduction and refinement), the prediction method of toxicity theory has a good prospect dependent on high-quality and comprehensive data sources [99].

In fact, the use of NAMs is beginning to result in an emerging consensus on how to use information from in silico, in vitro and targeted in vivo sources to assess the safety of chemicals. However, this methodology is being adopted very slowly for regulatory purposes. Recently, Ball et al. developed a framework incorporating in silico, in vitro and in vivo methods designed to meet the requirements of REACH, in which both hazard and exposure can be assessed [100]. REACH is a European regulation on the Registration, Evaluation, Authorization and Restriction of Chemicals in the European Union. The REACH initiative already prescribes that animal testing must be undertaken only as a last resort [101]. The framework allows a transparent and phased introduction of NAMs in chemical safety assessments and enables science-based safety decisions that provide the same level of public health protection using fewer animals, taking less time and using less financial and expert resource [100].

Different agencies also provide models and databases on the Internet. For instance, the European Chemicals Agency (ECHA) [102] published a practical guide on how to use alternatives to animal testing to fulfill regulatory requirements (also explaining the conditions that need to be fulfilled to use QSAR predictions), citing useful examples for good prediction practices based on commonly used and freely available QSAR software [103].

Currently, cosmetics safety evaluation has achieved quite good results. Most countries use the theoretical prediction method as their major evaluation method, followed by other safety evaluations. Accelerated advances in artificial intelligence and complex networks have also contributed to the innovation and development of theoretical toxicity prediction methods [104]. Through data fusion methods and the efficiency evaluation of chemical structure data, the prediction performance of computational toxicity may be improved. The future expansion of computational toxicity may pioneer studies on peptides and nanomaterials to find a reasonable format to simplify the complexity of the parameters. Moreover, an important issue of in silico models for toxicity prediction is how to learn with limited available data rather than training on a large amount of data. In view of the low popularity, accuracy and reliability of toxicity prediction models at present, a variety of toxicity prediction models with high accuracy and good reliability have been established for different toxicity endpoints to improve the performance of toxicity prediction models. The potential molecular descriptors or key molecular fragments related to toxicity have been obtained [105].

Lately, Daood et al. employed a data-driven QSAR modeling workflow to extensively enlarge the limited training data by revealing multiple targets involved in immunotoxicity. To do this, a data set of 6341 compounds was obtained from an HTS assay testing for the activation of the aryl hydrocarbon receptor (AhR) signaling pathway, a key event leading to immunotoxicity. Searching this data set against PubChem yielded 3183 assays, with testing results for varying proportions of these chemicals. In total, 100 assays were selected to develop QSAR models based on their correlations to AhR agonism. Moreover, 12 QSAR models were built for each assay using combinations of four ML algorithms and three molecular fingerprints. In addition, 20 assays were further developed based on QSAR model performance, and their resulting QSAR models showed a good prediction of potential immunotoxicants from external compounds. This study proves that large public toxicity data sets can be used to model immunotoxicity or other toxicity endpoints that have limited training data [106].

The intuitive factors that affect the modeling process and prediction performance include data availability, fast processing capacity, feature selection methods (such as molecular descriptors) and evaluation methods. In this regard, the desire to optimize the design of new drugs has led to the development of new in vivo experimental models, which represent cheaper and faster alternatives to the original models. Within these alternative models, the larvae model from the insect *Galleria mellonella* (*G. mellonella*) is gaining popularity since, unlike other non-mammalian models, it allows toxicity and anti-infective activity tests to be carried out since they survive at 37 °C [107]. Furthermore, the size of the larva allows for convenient inoculation with a specific dose of microorganisms [108].

The first toxicity test with *G. mellonella* dates to 1949 and was focused on the study of the variability in the toxic effect of a group of insecticides depending on their route of administration [109]. However, it was not until the early 2000s that the model was used to study the toxicity of compounds whose goal is to be administered to humans. There are studies that confirm that the immune response of these larvae is very similar to that of humans and rodents [110,111,112]. This is mainly because it consists of structural and passive barriers, as well as humoral and cellular responses [113].

Currently, *G. mellonella* larvae have been used as an infection model for bacteria, fungi and viruses [114,115]. Several studies aimed at determining the correlation between this new model and the traditional rodent model conclude that there is also a good correlation regarding the toxicity of the compounds studied in larvae and rodents [115,116].

The *G. mellonella* model was also used to test the relative toxicity of food preservatives. In total, 8 preservatives were tested, showing a high correlation between the LD50 found in larvae and that found in rats, which reinforces the usefulness of this model to determine the toxicity of different compounds [117]. Another study analyzes the effect of caffeine on these larvae since the characterization of its effect on insects could provide a better understanding of how this substance acts in mammals. The results show that these larvae metabolize caffeine, producing theobromine and theophylline. In addition, the results also indicate a correlation with the results obtained in zebrafish embryos, demonstrating once again the usefulness of the model for the in vivo experimentation of a great diversity of compounds [118].

Megaw et al. studied the toxicity of ILs in these larvae. This research is very interesting because ILs, as mentioned earlier, can be an ecological alternative to organic solvents. The study concluded that the 1-alkyl-3-methylimidazolium chloride ILs tested produce a toxic effect on larvae that is directly proportional to the length of the alkyl side chain [119].

Suay-García et al. studied the solubility of different solvents for application in in vivo toxicity tests of the *G. mellonella* larvae model, for which a protocol had to be designed since there are very few solvents described in the literature that have been studied for the administration of compounds to this insect [120], unlike other invertebrate models such as the nematode *Caenorhabditis elegans* or the fly *Drosophila melanogaster* [121]. In fact, the problem of solubility for toxicity tests on *G. mellonella* larvae has already been previously described by other authors [116,122].

The relative low cost of the *G. mellonella* model, the speed with which results can be obtained and the absence of ethical limitations make this model an ideal tool as a first step prior to testing in mammals, saving money and the time invested in assays if the compound turns out to be toxic or inactive in larvae [123]. However, it also has a series of drawbacks that should be considered. The absence of a standard protocol can lead to obtaining results that cannot be comparable or reproducible since there is great variability in the number of larvae used for each test, the observation time after the administration of the compound, the temperature at which the larvae should be maintained during the study, the injection volume, and the number of times each test should be repeated.

## 7. Conclusions

The 21st century is the golden age of the development of computational toxicology. Despite the great progress of biology, chemistry and computer science, there are still many computational methods to be explored, and there are also many existing issues to be repaired and solved. In this review, we defined the origin of toxicology and the development of ML and DL. We also outlined that QSAR methods are an effective tool for the rapid virtual screening of compounds with potential biological activity. Furthermore, this cheminformatics approach allows the prediction of other pharmacokinetic and toxicological properties directly related to activity to find safer and more effective drugs. These methods can also be extremely useful in drug repositioning, where we find thousands of commercial compounds, which will have most certainly endured toxicological assays, to be screened in a short time. With the support of the described theories and technologies, researchers began to widely apply them in the pharmaceutical industry, biology field and environmental chemistry.

The present review briefly introduces the related research and technological breakthroughs of toxicology in the environmental field. We also point out the present difficulties and expect that there will be more technological breakthroughs in the future. Finally, we believe that these obstacles will be overcome with the development and refinement of tools of application in this field.

## Figures and Tables

**Table 1:** Examples of available software and online platforms employed in QSAR.

Software	Main Features	Ref.
QSARPro	Performs group-based QSAR approach, establishing a correlation between chemical group variation at different molecular sites of interest and the biological activity.	[14]
MedChem Studio	Cheminformatics platform supporting lead identification and prioritization, de novo design, scaffold hopping and lead optimization.	[15]
McQSAR	Free program to generate QSAR equations using the genetic function approximation paradigm.	[16]
PADEL	Free software to calculate molecular descriptors and fingerprints.	[17]
Codessa	Uses quantum mechanics-derived descriptors to develop QSAR/QSPR models.	[18]
cQSAR	Program for interactive, visual compound promotion and optimization. It includes PD and PK parameters and can be linked to other modules for physicochemical and ADME.	[19]
MCASE	ML approach to automatically evaluate compounds/activity data set and identify the biophores. It then creates organized dictionaries of them and develops ad hoc local QSAR correlations.	[20]
SMIREP	System for predicting the structural activity of chemical compounds.	[21]
Alvascience	QSAR software package that uses in silico techniques to analyze chemical datasets and evaluate the physico-chemical and ecotoxicological properties of chemicals.	[22]

**Table 2:** Several in silico modeling methods.

Methodology	Definitions	Model Types	Limitations
Quantitative structure–activity relationships (QSARs)	Use molecular descriptors Predict chemical’s toxicity	Local and global QSAR, SAR, QSTR and QSPR	Requires large database, feature selection
Pharmacokinetic (PK), Pharmacodynamic (PD)	PK and PD models evaluate concentration at a given time and calculate effect at a given concentration, respectively	One-compartment models, two-compartment models	PK and PD parameters may be unavailable or inaccurate
Structural alerts (SAs), rule-based	Chemical structures associated with toxicity	Human-based rules, induction-based rules, pattern growth	SAs cannot provide insight into the biological pathways of toxicity
Read across (RA)	Predict unknown toxicity of chemical using similar chemicals with known toxicity	Analog approach, category approach, qualitative and quantitative RA	Use small datasets, accuracy depending on the number and choice of analogs, similarity metrics

**Table 3:** Chemical structures of groups associated with high reactivity or interferences in assays.

Chemical Structure	Group Name	Screening Liability
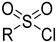	Sulfonyl chloride	Can metabolize, causing genotoxicity
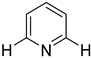	2,6-unsubstituted pyridine	Potential interference with cytochrome P450s due to metal ion coordination
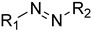	Azo	Potentially carcinogenic and mutagenic
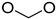	Acetal	Metabolically unstable due to acetal hydrolysis
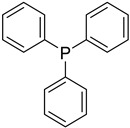	Triphenylphosphane	Produces DNA double-strand breaks (genotoxic) and human cell death effects (cytotoxic)
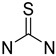	Thiourea	Metabolically unstable due to flavin oxidation Potential non-specific protein binding
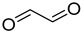	1,2-dicarbonyl	Metabolically unstable Potential toxicity due to mutagenicity
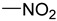	Nitro	Prone to reduction, yielding reactive species Potential hepatocarcinogen
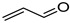	α,β-unsaturated carbonyl	Prone to reactivity by acting as a Michael acceptor
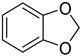	Methylenedioxy	Metabolically unstable due to acetal hydrolysis Prone to oxidation, yielding reactive quinones
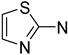	Aminotiazole	Potential toxicity
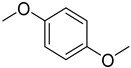	1,4-dimethoxybenzene	Very prone to oxidation, yielding reactive quinones
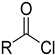	Chlorocarbonyl	Potential genotoxic impurity
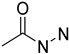	Acylhidrazide	Metabolically unstable due to acyl hydrolysis

**Table 4:** Computational toxicology in environmental research.

Year	Content	Method	Conclusion	Author
2012	A series of SMILES meant serial kernels and SVM were used to classify chemical toxicity in a toxicity database network (DSSTox)	SVM	The AUC values of DBPCAN data, NCTRER data, EPAFHM data, CPDBAS data and FDAMDD data are 0.950, 0.901, 0.740, 0.823 and 0.840, respectively	Cao et al. [78]
2018	Four quantitative toxicity data sets were used: LC50, LC50-DM, IGC50 and LD50. DNN, RF and GBDT are used to build the model	DNN RF GBDT ^a^	According to the coefficient r^2^ of four data sets, the fitting effect of the DNN is the best, and the results obtained are more accurate	Wu et al. [79]
2021	In a study on drug-induced chemical ototoxicity, 1102 ototoxic drugs and 1705 non-ototoxic drugs were collected. ML and DL algorithms were used to construct individual models and consensus models, and a structural characteristics analysis of ototoxic drugs was conducted	ANN SVM RF XGBoost ^b^ TCNN ^c^	The performance of the consensus model on the test set and external verification set is better than that of the single model, and the accuracy rates are 0.95 and 0.90, respectively	Huang et al. [80]
2021	An SVM and GA model was established on a large data set of 840 organic compounds to explore the toxicity prediction of chemicals to various fish	SVM GA ^d^	The decision coefficient r^2^ of the SVM model is above 0.70 on both the training set and testing set, which shows good prediction performance	Yu et al. [81]

^a^ GBDT: gradient boosting decision tree. ^b^ XGBoost: extreme gradient boosting. ^c^ TCNN: transformer convolutional neural network. ^d^ GA: genetic algorithm.

## Data Availability

No new data were created or analyzed in this study.
